# Microfluidic device for primary tumor spheroid isolation

**DOI:** 10.1186/s40164-017-0084-3

**Published:** 2017-08-07

**Authors:** Jiaojiao Zhou, Jimmy Su, Xiaotong Fu, Lei Zheng, Zhizhong Yin

**Affiliations:** 1 Department of Oncology, Johns Hopkins University School of Medicine, Baltimore, MD USA; 2 Euveda Biosciences Inc., Baltimore, MD USA

**Keywords:** Microfluidics, Spheroid, Organoid, Cell Culture, Primary Cell, Cancer

## Abstract

**Background:**

Traditional two-dimensional (2-D) monolayer cell culture is vastly different from in vivo physiological conditions, which can lead to inaccurate or insufficient data in areas where response and efficacy within humans are being investigated, such as drug discovery, pathology studies, etc. Misleading results arise from two main disadvantages of monolayer cell culture. First, after several passages, cell lines lose many features from their original in vivo state. Second, the morphology of cells cultured in a monolayer is much different from the cell morphology in three-dimensional (3-D) in vivo conditions, thus resulting in altered cellular function. Three-dimensional multi-cellular spheroids, on the other hand, are a better representation of in vivo physiological conditions while still retaining many of the in vitro cell culture advantages. Primary spheroids freshly isolated from tissue samples are especially ideal for cell-based assays by avoiding the two problems of 2-D monolayer cell culture.

**Methods:**

In this paper, we report a microfluidic device for primary tumor spheroid isolation. Pancreatic tumor samples from mice were used in the experiments.

**Results:**

We successfully isolated primary tumor spheroids from the pancreatic tumor samples and were able to maintain the spheroids in culture for up to two weeks.

**Conclusions:**

This novel microfluidic device may promote and advance the isolation of primary tumor spheroids for future drug testing and interrogation of tumor characteristics.

## Background

Two-dimensional monolayer cell culture in cell culture dishes (or flasks or plates) is still widely used in academic, pharmaceutical, and biotechnology research due to its availability in large volumes and easy-to-maintain procedures. However, traditional cell culture has some fundamental flaws that limit potential insights into true physiological responses. First, the cellular morphology of cells cultured in a monolayer is a far cry from cells’ normal morphology in vivo. Cells in most organisms, including humans, live in a complex 3-D environment surrounded by other cells (both of the same type or different types of cells) and extracellular elements. This different microenvironment can lead to vastly different cell behaviors, including proliferation, differentiation, metabolism, etc. [1]. Unsurprisingly, this can lead to errant results in assays based on 2-D monolayer cell culture. For example, cell-based drug screening in pre-clinical stages of drug discovery can lead to false drug leads and very costly failures in later stages of drug discovery. Ninety percent of the chemical compounds that pass through early stage screening fail in the costly clinical trial stages. Some of these failures can be attributed to the non-ideal cell culture system used in the early stages of drug screening [2, 3].

Three-dimensional cell culture, which is believed to be a better alternative to 2-D monolayer cell culture, has seen increasing utility and popularity over recent years. Different methods of 3-D cell culture, i.e., in different types of gels, in droplets, etc., have emerged as researchers continue to gain a better understanding of 3-D culture microenvironments [1, 4]. In addition, new technologies such as microfluidics allow for more precise and throughput methods for cell-based assays. For example, many kinds of organ-on-a-chip devices have been developed to better recapitulate physiological function and response of different organs in an organism [5]. Spheroids, in which different kinds of cells live together in a 3-D, roughly spherical shape, have also generated interest in research fields with the development of new technologies. Spheroids can be formed from single-cell suspensions in hanging drops suspended over microtiter plates [6], within hollow microspheres [7], or using specially-constructed microfluidic devices with specific designs, controlled flow, and other techniques [8–11].

Spheroids (also sometimes referred to as “organoids”) are especially important for cancer research as it presents a more realistic model of tumor tissue [12–15]. A number of previous research studies have demonstrated the ability to form spheroids from established cell lines within microfluidic devices [10, 11, 16]. However, the use of established cell lines in cell-based assays is limited by two main drawbacks: (1) the generation of immortalized cell lines alters the biology of the cells [17], and (2) after several passages, cells tends to lose their desired physiological features and thus their relevance to clinically-applicable [18]. Furthermore, spheroids formed from cell lines, even including multiple cell lines, lack the heterogeneous composition and physiological characteristics of primary cell and tissue samples.

The aforementioned disadvantages of 2-D monolayer cell culture and the use of established cell lines lead us to believe that spheroids isolated from primary tissue samples are the best model for many cell-based assays. This is especially true for cancer-related studies and anti-cancer drug development as 2-D models cannot fully capture the complexity of the cancer microenvironment [19]. In this paper, we present a novel microfluidic device capable of isolating spheroids directly from primary tumor samples. The device facilitates the separation of spheroids from single cells in the primary tumor sample, and the captured spheroids can be readily collected for further analysis and culture. We demonstrate that the isolated spheroids contain a heterogeneous population of cells, including KPC tumor cells, Emr1-expressing macrophages, and α-SMA-expressing fibroblasts. Finally, we verify that the isolated and collected spheroids remain viable and can be cultured in a Matrigel-supported environment for up to 2 weeks.

## Methods

### Cell culture and reagents

Immediately after euthanizing KPC (Kras^G12D^Trp53^R172H^;Pdx1-Cre) mice, the fresh primary pancreatic tumor tissue was resected and transferred into complete medium. The complete medium was composed of RPMI 1640 supplemented with 10% fetal bovine serum and 100 μg/ml penicillin and streptomycin. The collected tissue was washed three times with PBS and transferred to a petri dish. Then the tumor tissue was finely minced with a sterile scalpel and transferred into a sterile centrifuge tube with digestion medium. The digestion medium was prepared with complete medium containing Collagenase Type IV (Invitrogen, Life Technology) and Hyaluronidase (Sigma). The mixture of finely-minced tissue and digestion medium was incubated at 37 °C for 1 h in a shaker. After incubation, the digested mixture was allowed to settle for 5 min and the supernatant was discarded. The precipitate was strained through a cell strainer (100 μm, BD Biosciences) and resuspended in complete medium.

The collected spheroids were cultured in a polymerized mixture of growth medium and Matrigel Basement Membrane Matrix (BD Biosciences). The growth medium for the spheroids was composed of RPMI 1640, 10% fetal bovine serum, 1 mM sodium pyruvate, 2 mM l-glutamine, and 100 μg/ml penicillin and streptomycin. The growth medium was replaced every 2–3 days.

### Design and fabrication of the spheroid isolation device

The microfluidic chip was made using soft lithography techniques as previously described in [20, 21]. Briefly, photoresist SU8-2025 (Microchem, Inc) was spin-coated on a polished silicon wafer. After baking, the SU8 layer was then patterned with a transparent mask and developed to form the microchannel network mold. The chip with the microfluidic network was fabricated by casting polydimethylsiloxane (PDMS) onto the SU8 mold. The mold with PDMS was baked at 80 °C for 1.5 h. The PDMS was then peeled off from the mold, cut, and drilled to generate the inlet/outlet interface with the fluid control system external to the device. Finally, the chip was bonded to a glass cover slip and baked at 80 °C for about 5 h.

**Fig. 1:**
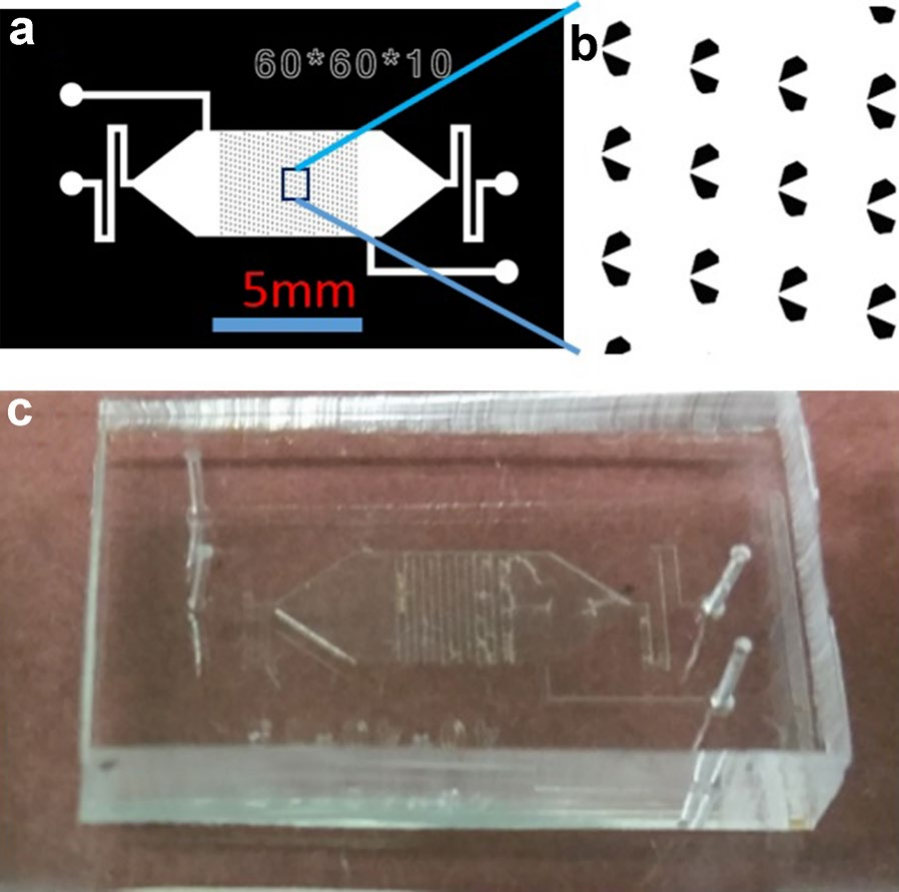
Design and fabrication of the spheroid isolation device. **a** Design layout of the chip. It has four inlets/outlets and a main chamber with hundreds of spheroid isolation units. **b** Close-up of the funnel-shaped spheroid isolation units. The *rows* of spheroid isolation units are slightly offset to promote efficient trapping of spheroids. **c** Picture of a real device

The chip has four inlets/outlets and a main chamber with hundreds of spheroid isolation units (Fig. 1). Spheroid isolation units are funnel-shaped with a big opening in the front and a small opening at the end. The size of the unit can be adjusted according to the size of the spheroid in concern. The small opening at the end is large enough to allow single cells to pass through while keeping any trapped spheroids in place. In our design, the typical size of the small opening is about 10–15 μ, and the big opening is 40–100 μ. The rows of the spheroid isolation units are in an alternating pattern to allow efficient trapping of spheroids. The height of the chamber is 50 μ.

### Sequencing of captured spheroids for Kras mutations and RT-qPCR of Emr1 and α-SMA

Total RNA from the captured spheroids was isolated with a RNeasy™ Micro kit (Qiagen), and cDNA was synthesized with the GoScript™ Reverse Transcription System (Promega). The primer set for PCR amplification of murine *Kras* codon 12 (exon 1) were as follows: *Kras*-F:5′ACTTGTGGTGGTTGGAGCTG-3′, *Kras*-R:5′-TGACCTGCTGTGTCGAGAAT-3′. The PCR products were then sequenced using the primer *Kras*-R for *Kras* mutation detection.

RT-qPCR was used to evaluate the mRNA expression of *Emr1* and *α*-*SMA*. Sets of primers were used as follows: *α*-*SMA*-F: 5′-GTCCCAGACATCAGGGAGTAA-3′, *α*-*SMA*-R: 5′-TCGGATACTTCAGCGTCAGGA-3′; *Emr1*-F: 5′-CCCCAGTGTCCTTACAGAGTG-3′, *Emr1*-R: 5′-GTGCCCAGAGTGGATGTCT-3′;*GAPDH*-F: 5′-TGGATTTGGACGCATTGGTC-3′, *GAPDH*-R: 5′-TTTGCACTGGTACGTGTTGAT-3′,

## Results and discussions

### Experimental procedure

The cell suspension was freshly prepared from the dissected primary pancreatic tumor of KPC mice. All the procedures were carried out gently; violent mixing, vortexing, and centrifuging were avoided in order to maintain the original native state of the spheroids.

**Fig. 2:**
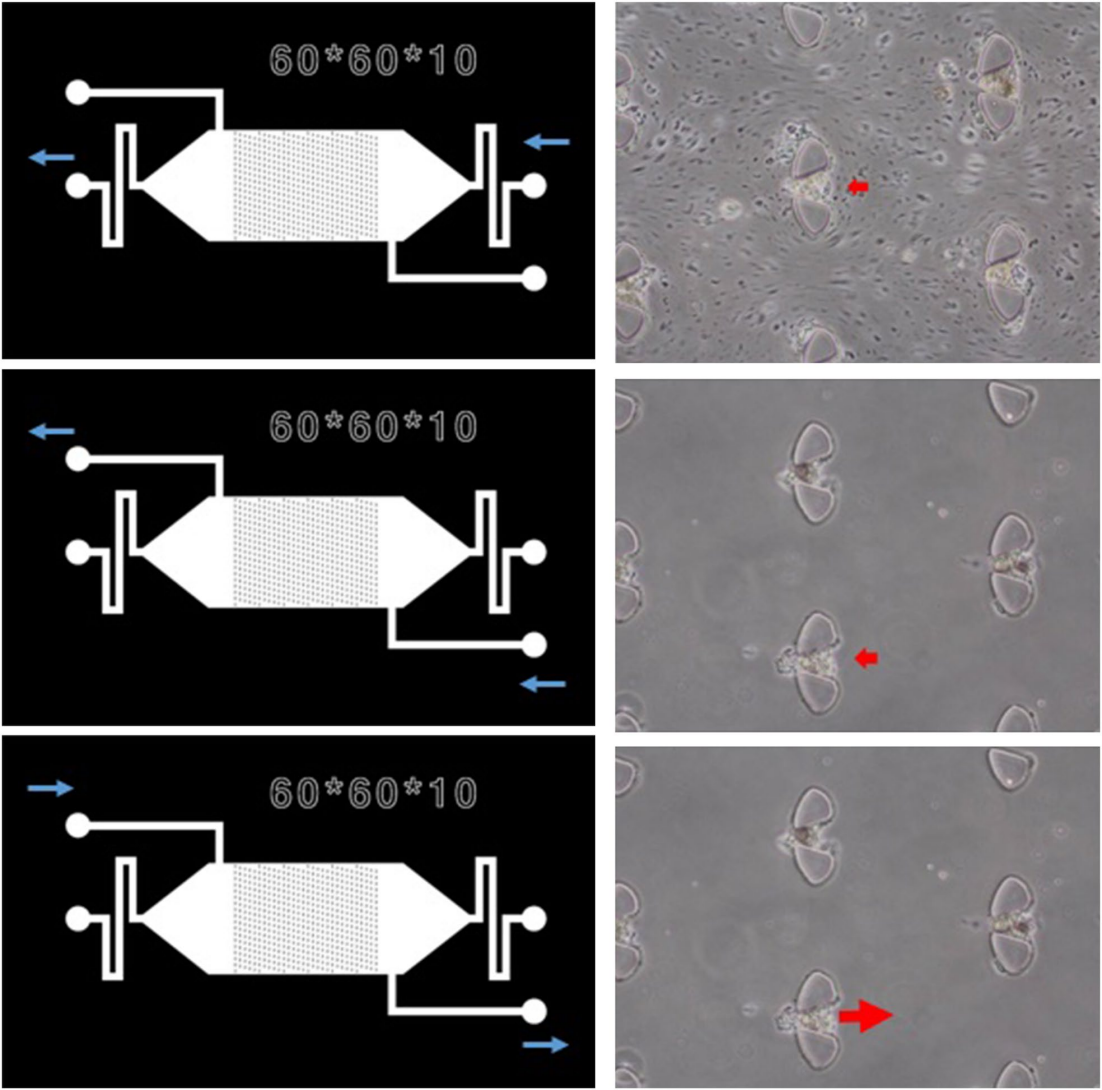
Work flow of spheroid isolation process using the device. *Top panel* cell suspension flows into the chip. *Spheroids* were captured by the trapping units. Single cells were not trapped by the tripping units as the opening is big enough for single cells to flow through. *Middle panel* the chip was washed with cell culture media to remove debris and excessive cells. *Bottom panel spheroids* were released from the chip through the bigger opening of the trap by reversing the fluid flow

The chip was primed with cell growth medium before the experiment to clear air out of the chip. Once both the cell suspension and the chip were prepared, the cell suspension was introduced to the chip through the cell inlet (Fig. 2). The cell suspension was allowed to flow through the chip driven by the pressure difference between the inlet and outlet. Appropriately-sized spheroids were captured by the spheroid isolation units (Fig. 2). The microfluidic chip was put under a microscope for observation. The flow of cell suspension was stopped when most of the spheroid isolation units contained trapped spheroids. Then cell growth medium was introduced into the chip through another inlet to wash out any unwanted debris. After the chip was washed clean, the flow was reversed and the trapped spheroids were released from the chip and collected in a tube. In our chip, there are more than 500 spheroid isolation units. Spheroid isolation efficiency of the chip depends on many facts, such as the size of the units, how many spheroids are presented in the cell suspension, as well as flow conditions. We were unable to take pictures of all the units. According to the pictures we have, around 50–60% spheroid isolation units can successfully capture spheroids. Trypan Blue was used to confirm the viability of trapped spheroid in separate experiment. A similar spheroid trapping device has been reported in [22] in which spheroids generated from an established cancer cell line were captured within a trap formed by PDMS posts within the device. This device has the added capacity to measure the dielectric properties of the trapped spheroids over time using electrical impedance spectroscopy; however, the throughput of the device is low (trapping only a single spheroid per device), and it is difficult to collect and culture the spheroids afterwards.

## Results

**Fig. 3:**
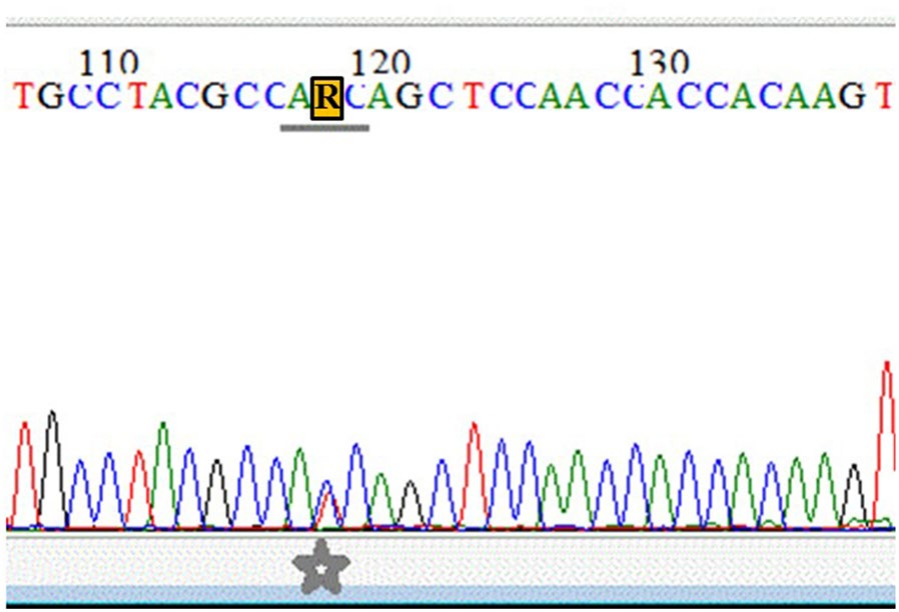
The trapped spheroids carried the *Kras* ^*G12D*^ point mutation. According to the sequencing map of the amplified *Kras* codon 12 (exon 1), both the A ***T*** C and A ***C*** C sequences are present at the site of mutation (the T-peak is equal to the C-peak). Therefore, the complementary sequence corresponds to G ***A*** T and G ***G*** T, which represents the mutated *Kras* ^*G12D*^ and the wild-type *Kras* sequences, respectively

To verify the composition of trapped spheroids, two experiments were conducted. First, we verified the existence of the KPC tumor cells in the spheroids by examining the mutational status of the *Kras* loci (Fig. 3). The sequencing result of amplified *Kras* codon 12 (exon 1) demonstrated that the isolated spheroids carried the Kras^G12D^ point mutation, which indicating the existence of the KPC tumor cells.

**Fig. 4:**
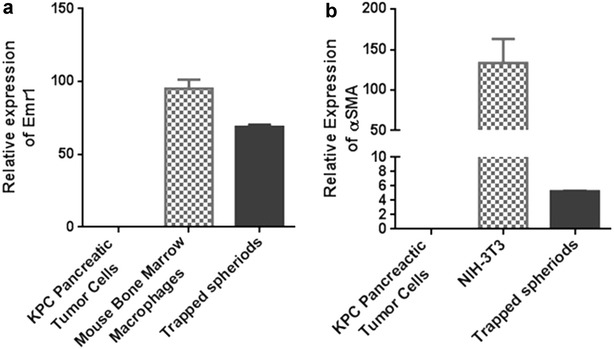
Mouse macrophage-specific marker Emr1 is highly expressed in the captured spheroids (**a**), and fibroblast-specific marker α-SMA is also moderately expressed in the captured spheroids (**b**). For Emr1 expression, KPC pancreatic tumor cells were used as a negative control, and murine bone marrow macrophages were used as a positive control. For α-SMA expression, KPC pancreatic tumor cells were used as a negative control, and murine NIH-3T3 fibroblasts were used as a positive control. The relative mRNA expression of Emr1 and α-SMA were measured by RT-qPCR

Then we did a PCR experiment to analyze other cell types in the spheroids and found that the macrophage-specific marker Emr1 was highly expressed in the captured spheroids (Fig. 4a). The fibroblast-specific marker α-SMA was also moderately expressed in the captured spheroids (Fig. 4b). These results indicate that at least two other types of cells are present in the spheroids: fibroblasts and macrophages. We believe the heterogeneous spheroid represents a better system than purified KPC tumor cells for drug testing and other tumor related studies, since it represents the actual tumor microenvironment more closely. For example, pancreatic tumor cells are sensitive to chemotherapy such as Gemcitabine, while a lot of studies demonstrated that both of macrophage and fibroblasts mediate gemcitabine resistance of pancreatic tumor cells [23, 24].

**Fig. 5:**
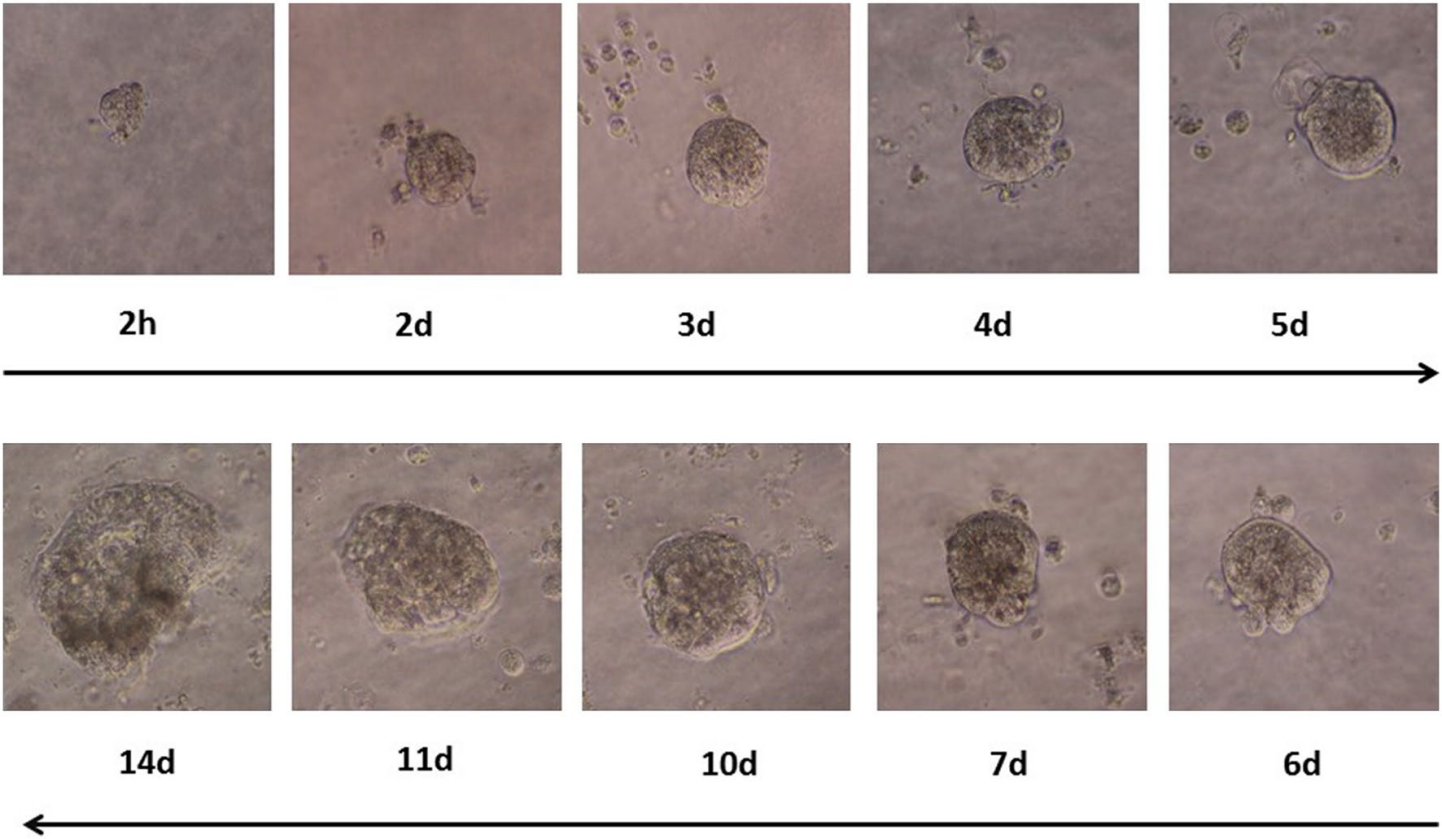
Culture and proliferation of a captured spheroid in the Matrigel polymerized growth medium for 14 days

Next, we tested the viability of the collected spheroids. The trapped spheroids were released from the chip, diluted in a mixture of growth medium and Matrigel, and quickly aliquoted into a 96-well plate at one spheroid per well before gelation. Then the viable spheroids were cultured at 37 °C in 5% CO_2_, and the growing spheroid colonies were monitored daily. Time-lapse microscopic monitoring of seeded spheroids in the first 48 h of culture revealed the proliferative capacity of the viable spheroids (Fig. 5). The proliferation was still robust after 14 days of culture (Fig. 5). The culture was stopped after 14 days since we deem 14 days is enough for most tests.

## Conclusions

In this paper, we successfully isolated primary tumor spheroids using a specially designed microfluidic device. In comparison to traditional 2-D monolayer cell culture, primary tumor spheroids have two major advantages: multi-cellular spheroids in 3-D formation preserve cells’ in vivo physiological condition, and primary cells from fresh tissue samples keep most features of cells in their original state. In addition, the microfluidic device allows for simple and high-throughput isolation of spheroids from resected tumors as well as easy visualization using an inverted microscope. The isolated spheroids can be cultured in a cell culture plate afterwards for further analysis. This novel microfluidic design may promote and advance the isolation of primary tumor spheroids for future drug testing and interrogation of tumor characteristics. The PCR results suggest that there were at least three different types of cells in the isolated spheroids: the KPC tumor cells, fibroblasts, and macrophages. The experimental studies and results presented provide us insight for future investigations involving the re-constitution of spheroids from purified primary cell cultures.

## Abbreviations

2-D: two dimensional
3-D: three dimensional
KPC: Kras^G12D^Trp53^R172H^,Pdx1-Cre
PDMS: polydimethylsiloxane
Emr1: epidermal growth factor module-containing mucin-like receptor 1
α-SMA: alpha smooth muscle actin
*Kras*: Kirsten rat sarcoma
